# Cladistic analysis of genotype data-application to GAW15 Problem 3

**DOI:** 10.1186/1753-6561-1-s1-s125

**Published:** 2007-12-18

**Authors:** Hsuan Jung, Keyan Zhao, Paul Marjoram

**Affiliations:** 1 Keck School of Medicine, Preventive Medicine, University of Southern California, 1540 Alcazar Street, CHP-220, Los Angeles, California 90089-9011, USA; 2 Program in Molecular and Computational Biology, University of Southern California, 1050 Childs Way, Los Angeles, California 90089-2910, USA

## Abstract

Given the increasing size of modern genetic data sets and, in particular, the move towards genome-wide studies, there is merit in considering analyses that gain computational efficiency by being more heuristic in nature. With this in mind, we present results of cladistic analyses methods on the Genetic Analysis Workshop 15 Problem 3 simulated data (answers known). Our analysis attempts to capture similarities between individuals using a series of trees, and then looks for regions in which mutations on those trees can successfully explain a phenotype of interest. Existing varieties of such algorithms assume haplotypes are known, or have been inferred, an assumption that is often unrealistic for genome-wide data. We therefore present an extension of these methods that can successfully analyze genotype, rather than haplotype, data.

## Background

In this paper we adopt a cladistic approach to association mapping. Such methods were first introduced by Templeton et al. [1], but other researchers have subsequently developed the ideas of that paper, or used other cluster-based approaches [2-4]. The methods are based upon haplotype, rather than single-nucleotide polymorphism (SNP)-by-SNP analysis. As the density of SNPs in current data sets increases, neighboring SNPs increasingly exhibit linkage disequilibrium (LD). A SNP-by-SNP analysis ignores this property, whereas a haplotype-based analysis directly exploits it.

However, the fact that current cladistic analysis methods act upon haplotype rather than genotype data also introduces a problem. Data are increasingly being collected for large numbers of SNPs, frequently via a SNPchip for which data on hundreds of thousands of SNPs might be collected. For such data, haplotype phase information is typically unavailable. The common approach to dealing with this (in a multi-locus analysis) is to infer haplotype phase and then perform an association study on these inferred haplotypes. However, the inference of haplotype phase is highly computationally intensive, with the better algorithms proving unable to infer phase in data sets containing SNPs on the order of thousands, rather than tens or hundreds. Such an approach is therefore completely impractical in large data sets (although we note, with interest, the recent development of fastPHASE [5]).

To circumvent this issue we propose a *greedy *approach in which, whenever pairs of genotypes are compared, the haplotypes of those individuals are assigned in a deterministic way so as to maximize the measure of haplotype similarity between those individuals. We then follow this with a cladistic analysis based upon CLASS, a cladistic association method used in, for example, Zhao et al. [6]. Our greedy assignment of haplotype phase has the merit of being computationally very simple. Thus, it allows the use of cladistic methods such as those above on genotype data without introducing an extreme computational burden. On the flip side, the method is heuristic, and therefore very unlikely to recover the true phase information, which may cause loss of power. Given the difficulty of using other haplotype estimation approaches, it is interesting to explore whether a computationally efficient, but naïve approach allows progress to be made. We do this via an analysis of the simulated (Problem 3) GAW 15 data. We show that a cladistic approach based upon greedy haplotype phase information performs quite well.

## Methods

Suppose we have data for *N *SNPs on *M *individuals. Let *L*(*i*) denote the location (in kb) of SNP *i*. Let *g*_*k*, *i *_denote the genotype data for individual *k *at SNP *i*, where *g*_*ki *_is defined as the number of copies of the minor allele at this locus. (Our methods also generalize naturally to microsatellite data.) We proceed from left to right along each chromosome, assessing similarity between pairs of genotypes around each SNP. Suppose we are considering a pair of individuals *j*_1_, *j*_2 _in a region centered around SNP *I*, on chromosome *C*. Intuitively speaking, at each location *I*, our algorithm calculates a similarity measure equal to the maximum possible haplotype shared length around *I*. More formally, we define a function *f*_*i*_(*j*_1_, *j*_2_) as: 2 if g_*j*1, *i *_= g_*j*2, *i*_; 1 if |g_*j*1, *i*_-g_*j*2, *i*_| = 1; and 0 if |g_*j*1, *i*_-g_*j*2, *i*_| = 2. *f*_*i*_(*j*_1_, *j*_2_) simply counts whether both, one, or neither of the pairs of haplotypes for *j*_1_, and *j*_2 _can match at SNP *i*. We wish to stop recording shared lengths on a given haplotype as soon as a mismatch is found, so we further define *F*_*i*_(*j*_1_, *j*_2_) as

$$F_{i}(j_{1},j_{2})=\{\begin{array}{ll} min\{f_{i}(j_{1},j_{2}),F_{i-1}(j_{1},j_{2})\} & if\;i>I,\\ f_{i}(j_{1},j_{2}) & if\;i=I,\\ min\{f_{i}(j_{1},j_{2}),F_{i+1}(j_{1},j_{2})\} & if\;i<I. \end{array}$$

For each pair of individuals *j*_1_, *j*_2 _we define the similarity around *I *as *S*_*I*_(*j*_1_, *j*_2_), where

$$\begin{array}{c} S_{I}(j_{1},j_{2})=\sum_{i=I+1}^{C(R)}F_{i}(j_{1},j_{2})(L(i)-L(i-1))\\ +\sum_{i=C(L)+1}^{I}F_{i-1}(j_{1},j_{2})(L(i)-L(i-1)). \end{array}$$

*C*(*L*) [*C*(*R*)] denotes the left- [right-]most SNP on chromosome *C*. We explored a range of other haplotype similarity measures, with similar results (not shown).

We make several observations about this procedure. First, it is very quick to compute. Second, at each location, it calculates a similarity *S*_*I*_(·,·) based upon phasing each pair of genotypes such that the possible haplotype sharing between those two genotypes is maximized, and this is performed independently across all pairs of individuals. This is an approximation, in that our measure of identical-by-state shared length must be greater than or equal to that which is the case for the (unobserved) true haplotypes. In principle, we might calculate the assignment of phase that maximizes the total similarity score across all pairs of individuals, but this would be highly computational intensive, and completely intractable for data sets of the dimensions that are now becoming typical in genome-wide studies. Third, we are appealing to the intuition that failure to explicitly allow for dependencies across individuals when assigning haplotype phase will not introduce too much noise into the analysis, but will allow us to efficiently exploit cladistic analysis techniques that are known to perform well in haplotype-based contexts. We now give further details of our analysis.

At each SNP *I*, we form an *M *× *M *distance matrix using the reciprocals of the similarity measures *S*_*I*_(·,·). We then apply a hierarchical agglomerative clustering algorithm to construct a tree relating all individuals in the sample. Next, we use an iterative scheme to break this tree into a number of clusters (clades). Suppose that the current iteration has the tree broken into *c *clusters. At each step we explore all possible points at which the tree can be further broken. A *p*-value for each potential breakpoint is calculated by considering the clusters that exist after breaking the tree at this point. We define a factor variable, *W*, that takes a single value for all individuals within each cluster, but whose value differs across clusters. We then apply a Kruskal-Wallis non-parametric analysis of variance to both the new topology, with *c *+ 1 clusters, and the old topology, with *c *clusters, to see whether *W *better explains the phenotype of interest. Assuming at least one potential breakpoint results in a *p*-value that is smaller than that which was obtained from the representation with *c *clusters, we accept the breakpoint with the smallest such *p*-value. We iterate this process until no decrease in *p*-value is obtained. We then record the *p*-value obtained at this locus and repeat the scheme at every other locus. The intuition here is that if we are at a location near a mutation that influences the phenotype, it is likely that we will be able to break the tree into a number of pieces that explain the phenotype (with each cluster corresponding, perhaps, to a unique occurrence of that mutation).

This procedure results in a *p*-value for each SNP. It remains to assess the significance of the results. We expect markers near a trait locus will have small *p*-values. A traditional way to determine a significance cut-off for these *p*-values is to use a permutation scheme. For example, for a data set of interest, we create 100 new data sets in which we maintain the genotype data but permute phenotypes across individuals. We then analyze these data sets using our method and record the value of the lowest *p*-value observed in each case. A variety of other permutation tests are possible, depending on the hypothesis one wishes to test. It is common practice to follow a procedure such as the one we use here in order to attest the significance of the smallest *p*-value observed in the region under consideration (in our case, a chromosome, although one could perform a genome-wide permutation test if desired). We use the set of lowest observed *p*-values to define a significance cut-off for the original, observed data. Such a scheme is a computationally intensive method to employ. While the permutation test for a single replicate can be completed in about 12 hours, and is this highly tractable for real applications, use of the permutation test across all 100 replicates takes around 50 days per chromosome. Thus, we present permutation results only for chromosome 18 (the case in which significance of results is most in doubt – see below) [7]. For other analyses, we focus solely on a presentation of results showing the mean distance between the trait locus and the SNP with the smallest *p*-value (see Results and Discussion).

A further complication is that of computational efficiency. While our method, which is implemented in C code, is able to analyze samples of a few hundred individuals in around 10 minutes, it would prove impossible to analyze the full set of cases and controls, across all 100 replicates in the time available. Instead, we chose to implement the following scheme. For each analysis of a given chromosome, for a particular phenotype of interest, we construct 10 data sets consisting of 100 'cases' and 100 'controls' (sampling without replacement). The definition of 'case' and 'control' depends on the phenotype of interest, and is given in the Results. We analyze each of these 10 data sets, record the *p*-value for each locus in each analysis, and report the average *p*-value across the 10 analyses as the final 'score' for that locus.

Due to space requirements, the issue of missing values is not directly examined in this paper. However, it would be relatively straightforward to extend our methods using schemes that impute missing values. Alternatively, we could adjust the haplotype similarity measure *f*_*i*_(*j*_1_, *j*_2_) so that it includes a score, perhaps based upon allele frequencies at the locus, for genotypes with missing values at a given locus.

## Results and Discussion

Given the limitations of space, we have focused on a number of regions, and phenotypes, motivated by the knowledge of the answers. Specifically, we look at chromosomes 6, 11, 18, on which we expect to find signals (we performed analyses with the answers known to us), and chromosome 3, on which there should be no signal. We give details of each analysis, and present output for Replicate 1 of the GAW15 data to illustrate the behavior of our method. Then, in order to get an indication of power of the method, we look at behavior across all 100 replicates for each of these cases. In all cases we have used genotypes formed from the standard SNP data, (STR, and the dense SNP map on chromosome 6, have been excluded from the analysis). While we do not present more detailed results of other analyses tried upon Replicate 1, our analysis found no other strong signals. We believe this is largely because the other simulated traits largely involve effects due to interactions.

### Chromosome 6

We use the full set of the cases as well as the panel of 2000 control samples. Parents of cases were excluded. We use rheumatoid arthritis (RA) affection status as the binary phenotype of interest.

### Chromosome 11

The full population of cases (only) were used. IgM level was used as the phenotype. As described above, ten sets of sub-samples were analyzed, with results being averaged across sub-samples. Note that here the phenotype is continuous. Cases correspond to high values, while controls are low valued. (Our method is applicable to either discrete or continuous phenotypes.)

### Chromosome 18

**Figure 1:**
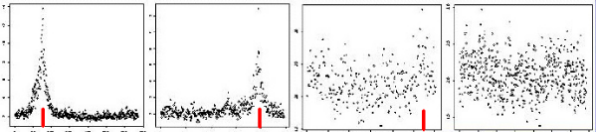
**Results of analysis of chromosomes 6, 11, 18, and 3 (left to right)**. The x-axis represents position along the chromosome (for convenience, markers are plotted as if equally spaced). The y-axis gives the -log *p*-value for association at each locus on that chromosome. The trait locus position marked with red line.

Here we analyzed just the case individuals. Anti-cyclic citrullinated protein (CCP) level was used as the phenotype. Cases were ranked according to anti-CCP level. Ten sub-samples of size 200 were then formed by sampling 100 'high' individuals with anti-CCP level = 210, and 100 'low' individuals with (anti-CCP level ≤ 20). No signal was seen (results not shown). However, when we restricted the analysis only to cases with a DR status of '3' we uncovered a signal, shown in Figure 1 and Table 1.

**Table 1:** Summary results across all 100 replicates for four chromosomes

Analysis	Range of log(*p*-values)	Mean distance from true locus (kb)^a^
Chromosome 6	[-34.8, -15.3]	19
Chromosome 11	[-17.81, -9.75]	31
Chromosome 18	[-5.69, -3.47]	5550
Chromosome 3	[-3.97, -3.34]	NA

^a^Distance between the functional locus and the locus corresponding to the smallest *p*-value.

### Chromosome 3

We also wished to analyze a region in which we did not expect to find a signal. Thus, we performed an analysis of chromosome 3 in which all details are the same as those given for chromosome 6 above.

In Figure 1 we show illustrative results, for chromosomes 6, 11, 18, and 3 (left to right in the figure), in this case for Replicate 1. SNPs are indexed on the x-axis, with the associated (-log) *p*-value plotted on the y-axis. We see clear signals (i.e., peaks) at the correct locations in chromosomes 6 and 11 (although only one signal is detected on chromosome 6). On chromosome 18 the signal is much less clear (being qualitatively similar to that seen for chromosome 3), but we note that the smallest *p*-value (i.e., highest -log *p*-value) is obtained very close to the correct location.

In order to assess power we present results across all 100 replicates in Table 1. We report the range of values observed over the 100 replicates for the smallest log *p*-value associated with any SNP, as well as the distance between the SNP with the smallest *p*-value and the trait locus. The former is an indication of significance of results; the latter is an indication of accuracy. We see that for chromosomes 6 and 11, *p*-values are very small, and the SNP with the smallest *p*-value is very close to the functional locus. Presumably, a permutation test would reveal them to be significant in each replicate (cf. permutation test results for chromosome 18; we have also checked that this is true for a permutation of data for these chromosomes on Replicate 1). The results for chromosome 18 are less clear. *p*-Values are not particularly small, and if we assume that each replicate leads to significant results we obtain a large average distance between the SNP with the smallest *p*-value and the trait locus. With this in mind, we conducted a comprehensive permutation test on chromosome 18. For each replicate we create 100 data sets in which we permute the phenotype (anti-CCP level) of all individuals within the sampled population, and then perform an identical analysis to that described for chromosome 18. If we then look only at replicates in which the lowest *p*-value for the unpermuted data was lower than the lowest *p*-value for all 100 permuted data sets, corresponding to an estimated *p*-value less than 0.01 before correction for multiple comparisons across chromosomes, we find that we locate the correct marker on 44 out of 65 such replicates. However, the average distance between the SNP with the smallest *p*-value and the trait locus across all 65 such replicates is still high (at 2860 kb), so our method is clearly not performing that well in this case (where the signal is substantially weaker than on chromosomes 6 and 11). Insistence upon a smaller estimated *p*-value (which would require a larger permutation test) may improve performance, but was prohibited by time constraints.

## Conclusion

The results in this paper demonstrate that a greedy method such as ours, in which maximal haplotype sharing is used as a proxy for actual (unobserved) haplotype sharing, offers the potential to reap some of the advantages of traditional haplotype-based methods while simultaneously avoiding much of the computational burden. Our method provides a quick way of analyzing genome-wide data in the absence of phase information. In other words, we sacrifice power for speed. As such, it might be used as part of preliminary data analysis, and used to focus on regions to subject to later, more detailed analysis. It should be noted that the GAW15 Problem 3 simulated data do not present the ideal platform to test the limits of our method. Simple locus-by-locus tests typically also find the signals we find (results not shown), and the simulated SNPs are too widely spaced for much LD to be present. Furthermore, our methods are designed primarily for unrelated population-based samples, rather than a situation in which some pedigree information is known. On chromosome 6, the only region in which dense SNPs are available, the signal is so strong that virtually any method will find it.

A major future focus of our work will be the detection of effects due to interactions. It should be possible to capture interaction between a factor-level exposure variable and a locus of interest within the iterative process in which we break the tree topology constructed using a particular locus and then assess fit. We might simply include the additional factor and potential interactions in the Kruskal-Wallis test that is used to assess *p*-values for each possible break of the tree. Assessing interactions between SNP loci will be less straightforward, and it will be subject to the usual difficulties involved in exploring large numbers of possible interactions [8].

Our method is deliberately 'naïve'. The goal is to simplify the analysis without losing the ability to detect signal. Thus, we speed the method such that we can perform a rapid 'haplotype'-based analysis of genome-wide data. This represents an example of a growing move away from exact, to more approximate methods, driven by the fact that the size and complexity of modern data sets often prohibits exact analysis in finite time for complex methods [9]. The final degree to which such a move is necessary in a genome-wide association study remains to be seen.

## Competing interests

The author(s) declare that they have no competing interests.
